# Identification of Novel PPARγ Partial Agonists Based on Virtual Screening Strategy: In Silico and In Vitro Experimental Validation

**DOI:** 10.3390/molecules29204881

**Published:** 2024-10-15

**Authors:** Yu-E Lian, Mei Wang, Lei Ma, Wei Yi, Siyan Liao, Hui Gao, Zhi Zhou

**Affiliations:** School of Pharmaceutical Sciences, Guangzhou Medical University, Guangzhou 511436, China

**Keywords:** PPARγ partial agonists, virtual screening, natural product library, TR-FRET competitive binding assay, podophyllotoxone

## Abstract

Thiazolidinediones (TZDs) including rosiglitazone and pioglitazone function as peroxisome proliferator-activated receptor gamma (PPARγ) full agonists, which have been known as a class to be among the most effective drugs for the treatment of type 2 diabetes mellitus (T2DM). However, side effects of TZDs such as fluid retention and weight gain are associated with their full agonistic activities toward PPARγ induced by the AF-2 helix-involved “locked” mechanism. Thereby, this study aimed to obtain novel PPARγ partial agonists without direct interaction with the AF-2 helix. Through performing virtual screening of the Targetmol L6000 Natural Product Library and utilizing molecular dynamics (MD) simulation, as well as molecular mechanics Poisson–Boltzmann surface area (MM-PBSA) analysis, four compounds including tubuloside b, podophyllotoxone, endomorphin 1 and paliperidone were identified as potential PPARγ partial agonists. An in vitro TR-FRET competitive binding assay showed podophyllotoxone displayed the optimal binding affinity toward PPARγ among the screened compounds, exhibiting IC_50_ and ki values of 27.43 µM and 9.86 µM, respectively. Further cell-based transcription assays were conducted and demonstrated podophyllotoxone’s weak agonistic activity against PPARγ compared to that of the PPARγ full agonist rosiglitazone. These results collectively demonstrated that podophyllotoxone could serve as a PPARγ partial agonist and might provide a novel candidate for the treatment of various diseases such as T2DM.

## 1. Introduction

The prevalence of type 2 diabetes (T2D), a metabolic disorder, is projected to escalate into a significant global public health concern within the next three decades. There were an estimated 529 million people living with diabetes in 2021 (96% were T2D), and that number will reach 1.31 billion by 2050 [1]. The occurrence of type 2 diabetes is attributed to reduced sensitivity of insulin-sensitive tissues towards insulin, resulting in diminished glucose uptake (GU) and subsequent hyperglycemia [2]. PPARγ has been extensively studied for its ability to mediate adipocyte differentiation in response to energy surplus and effectively regulate plasma glucose levels. Based on its pharmacological properties, PPARγ has been recognized as one of the most efficient targets for anti-diabetic drug discovery and development [3,4]. The activity of PPARγ is regulated by agonists, and the interaction between PPARγ and its ligands serves as a pivotal step in modulating PPARγ activity, with the structural characteristics of PPARγ playing a crucial role in this binding process [5].

**Figure 1:**
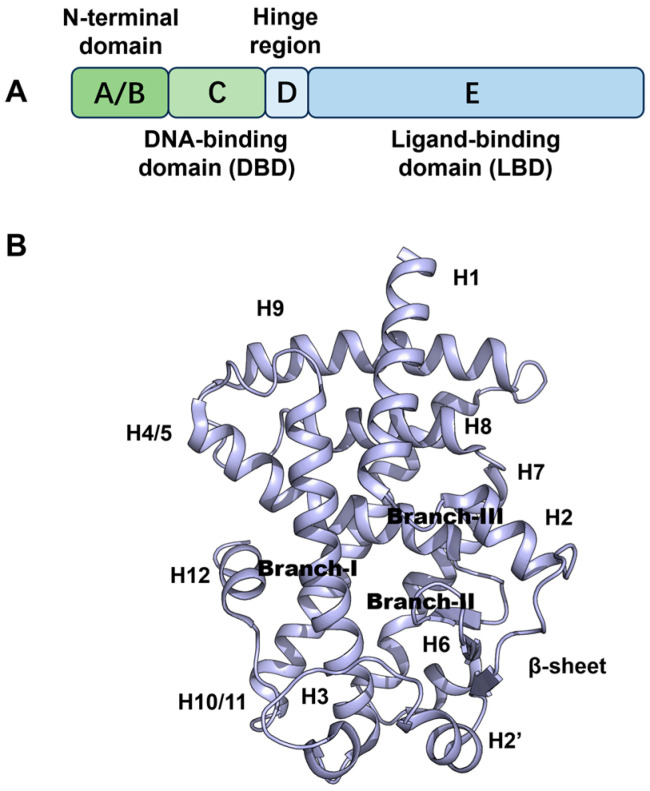
(**A**) Primary structure of PPARγ; (**B**) crystal structure of PPARγ (PDB code: 8DK4).

Structurally, PPARγ comprises distinct functional domains, including an N-terminal transactivation domain containing an activation function (AF1), a highly conserved DNA-binding domain (DBD) and a C-terminal ligand-binding domain (LBD) containing a ligand-dependent transactivation function (AF2) [6]. The structure of the PPARγ ligand-binding domain (LBD) is composed of 13 α-helices and four stranded β-sheets (Figure 1). The ligand-binding pocket has three branches and has been described as a large Y- or T-shaped cavity. Branch-I is hydrophilic in nature, and is formed by helices H3, H5, H11 and H12. Branch-I is located proximal to helix H12, which forms a critical part of the activation function-2 (AF2) coregulator binding surface. In contrast, Branch-II exhibits a hydrophobic character, formed by helices H2’, H3, H6 and H7 and a β-sheet region. Branch-III consists of both hydrophobic and hydrophilic regions, surrounded by a β-sheet as well as helices H2, H3 and H5 [7]. The LBD of PPARγ has several regulatory functions [8]. Depending on the specific ligand, agonists activate PPARγ, leading to conformational changes in the ligand-binding domain and subsequently inducing the transcription of distinct target genes, thereby enhancing insulin sensitivity. Some PPARγ agonists are prescribed for the management of T2D, such as rosiglitazone and pioglitazone [9]. The synthetic agonist rosiglitazone stabilizes helices H3 and H12, which constitute the AF2 site, subsequently inducing coactivator recruitment. The binding of rosiglitazone to the ligand-binding domain (LBD) effectively inhibits CDK5-mediated phosphorylation of Ser245. Reduced Ser245 phosphorylation alters the expression of a subset of genes with regulatory functions in metabolism; for example, it increases expression of the insulin sensitizing genes adipokine and adiponectin [10]. Due to the potential side effects of PPARγ full agonists, such as weight gain, fluid retention, vascular events and bone fractures [11,12], the European Marketing Authority recommended removal of rosiglitazone from the European market in 2010 [13]. However, partial PPARγ agonists MRL-24, nTZDpa and amorfrutin 1 display similar anti-diabetic effects to rosiglitazone due to their ability to block PPARγ-Ser245 phosphorylation as effectively as rosiglitazone while only moderately inducing the expression of PPARγ target genes involved in adipocyte differentiation [14]. In addition, these partial agonists do not contact helix H12 but rather stabilize helix H3 and the β-sheet region of the binding pocket of the PPARγ LBD [15]. Consequently, partial agonists exhibit hypoglycemic effects without causing severe side effects. In recent years, inverse agonists which decrease expression of PPARγ-controlled genes and antagonists which maintain the basal transcriptional output of PPARγ have emerged as safer alternatives to full agonists [16,17]. These principles and the associated structural insights offer a novel and rational approach for the development of effective PPARγ modulators in the pursuit of anti-diabetic drug discovery [5,6,18].

Natural compounds remain pivotal in drug discovery due to their extensive chemical diversity and potential therapeutic benefits. Historically, natural products, including plant extracts, have been significant sources of bioactive compounds [19,20]. In order to identify suitable candidates from natural products, our efforts focused on rapidly seeking new PPARγ partial agonists for anti-diabetic drug discovery. Thus, the present study’s aim is (a) virtual screening of the Targetmol L6000 Natural Product Library to search for potential hits that function as PPARγ partial agonists with the ability to block cyclin-dependent kinase 5 (CDK5)-mediated phosphorylation of PPARγ-Ser245 in the absence of classical transcription activity of PPARγ via the AF-2 helix “lock” mechanism; (b) further examination of the binding stability of the system utilizing molecular dynamics and molecular mechanics Poisson–Boltzmann surface area (MM-PBSA); (c) testing the PPARγ binding affinity and agonistic activity of the selected hit. Taken together, our present study aims to combine computational approaches with in vitro experimental validation, thereby constituting a logical and robust workflow for the identification of promising candidates targeting PPARγ.

## 2. Results

### 2.1. Docking Validation, Virtual Screening and Molecular Docking

To validate the docking protocol, we redocked the partial agonist (VSP-51-2) obtained from co-crystalized PPARγ complex (PDB code: 8DK4) which yielded a score of −10.96 kcal/mol. The docking pose was basically aligned with the co-crystal ligand, proving the reliability and validity of this methodology. Virtual screening of Targetmol L6000 Natural Product Library (containing 4320 compounds) was carried out using the Autodock Vina software [21]. The docking scores and structures of the top four compounds from virtual screening are listed in Table 1. The docking scores of the four highest-binding compounds ranged from −10.68 to −10.04 kcal/mol. Compared to the original ligand VSP-51-2, the binding affinities of these compounds were generally lower, with the top binding compound endomorphin 1 producing a 0.28 kcal/mol lower binding score than VSP-51-2. Nevertheless, the affinity scores were highly negative, suggestive of favorable binding. We further evaluated the interactions between the ligands and PPARγ (Table 1). These four ligands interact with crucial structural residues through a hydrogen-bonding network with PPARγ other than those involved in classical agonism, as defined by residues shaping the activation function surface 2 (His323, Tyr473 and His449) [15]. Taken together, these four compounds exhibited favorable characteristics as candidate compounds for further exploration. In summary, based on the obtained docking scores and binding conformation, the resulting docked structures can serve as initial models for subsequent molecular dynamics (MD) simulations.

### 2.2. Stable Assessment of MD Simulation

The stability of these crucial binding interactions could not be fully explained by the static nature of molecular docking, given the flexibility of the residues and the corresponding fluctuations in secondary structure. To overcome these limitations, we employed MD simulations to effectively assess the stability of the protein−ligand complexes and apo protein [22]. The average values of the Root Mean Square Deviation (RMSD), Root Mean Square Fluctuation (RMSF), Radius of Gyration (RG) and Solvent-Accessible Surface Area (SASA) of the five systems in the last 50 ns of MD simulations are provided in Table S1.

**Figure 2:**
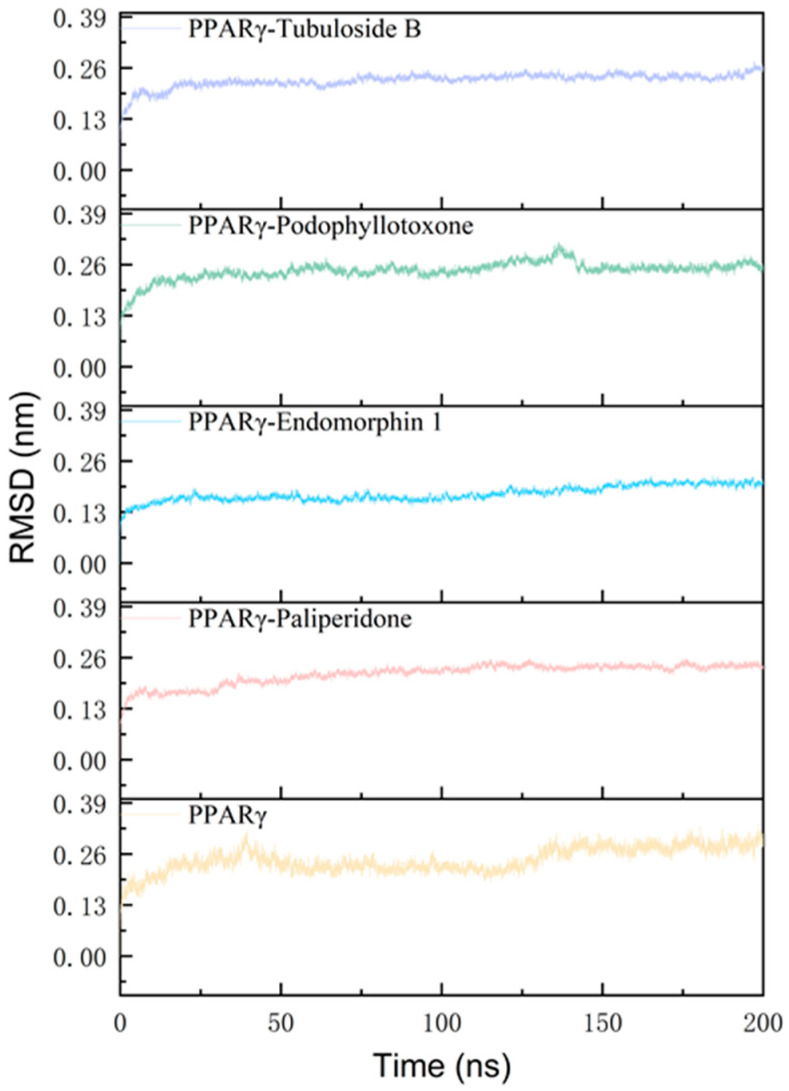
Analysis of RMSD of tubuloside b-, podophyllotoxone-, endomorphin 1-, paliperidone-bound and apo PPARγ.

RMSD evaluates the binding state and stability of the systems, with lower values indicating more stability in the system [22]. The RMSD of the protein backbone from the crystal structure of PPARγ are presented in simulation time (Figure 2). RMSD values tended to be stable after 150 ns, and the last 50 ns was selected for data analysis. Upon achieving equilibrium, the average RMSD of the backbone atoms fitted to the initial structure were 0.28, 0.24, 0.25, 0.20 and 0.24 nm for the tubuloside b-, podophyllotoxone-, endomorphin 1-, paliperidone-bound and apo PPARγ, respectively (Table S1). The apo protein exhibited a significantly higher RMSD value in comparison to the ligand-bound protein, thereby suggesting that the presence of these ligands contributes to enhancing the stability of the protein–ligand complexes. In conclusion, the overall RMSD values of the four systems ranged between 0.10 and 0.35 nm, indicating the stability of each system.

RMSF indicates the flexibility of residues in the systems. A higher RMSF value indicates a more loosely bonded structure with turns, bends and coils, whereas a lower RMSF value suggests a rigid secondary structure [23]. The RMSF values of the backbone atoms within the protein structure are depicted in Figure S2. The RMSF values of residues in four complexes fluctuated within the range of 0.05–0.60 nm, indicating overall stable dynamics. The RMSF values of the residues comprising the core structure remained consistently low across each system. The most significant differences were within the residues of Ala235–Ser245 (H2-H2′ loop) and Asp260–Lys275 (Ω loop) in PPARγ. Residues at helix H9 appeared to have the greatest fluctuation in apo protein compared with other ligand-bound systems. Overall, the average RMSF values of each system showed less deviations and suggested the overall stability.

The protein’s compactness relative to its backbone was determined by calculating the RG value. It was observed that throughout the simulation, the PPARγ protein exhibited a consistently compact secondary structure (Figure S3). The apo PPARγ tended to fluctuate around its initial conformation throughout the simulation, whereas the ligand-bound PPARγ appeared to become more compact over time. SASA can be calculated to assess the packing and stability of complexes during MD simulations. The SASA analysis of the PPARγ protein was in the range of 140–160 nm^2^ (Figure S4). In conclusion, both SASA and RG analyses confirmed the stability of the five systems.

### 2.3. Analysis of the Binding Energy

The calculation of binding energy (∆*E*_bind_) between the receptor and ligand is currently a commonly used and effective method for calculating binding affinity [24]. The MM-PBSA method allows a more accurate consideration of solvation contribution to the protein−ligand binding [25]. Therefore, in this study, we conducted the binding energy calculations between PPARγ and tubuloside b, podophyllotoxone, endomorphin 1 and paliperidone complexes, respectively. By comparing and analyzing the binding energy calculation results of all protein−ligand complexes (Table 2), it can be revealed that the binding energy of PPARγ with tubuloside b was the highest (−45.18 kcal/mol). This indicated that tubuloside b has higher binding affinity for PPARγ than the other three compounds, followed by endomorphin 1 (−38.86 kcal/mol), podophyllotoxone (−30.15 kcal/mol) and paliperidone (−28.08 kcal/mol). The obtained binding energy results convincingly demonstrated that these four compounds exhibit high binding affinities to PPARγ, making them suitable candidates targeting PPARγ.

Regarding the energy contribution of ∆*E*_bind_ (Table 2), we decomposed the energy to explore the details of the interactions between binding partners. ∆*E*_bind_ was decomposed into four components, namely van der Waals interactions (∆*E*_vdW_), electrostatic interactions (∆*E*_elec_), polar solvation contributions (∆*G*_polar_) and nonpolar solvation contributions (∆*G*_nonpolar_) [26]; see Equation (1) in the Section 3.4 for details on the decomposition. The van der Waals interaction (∆*E*_vdW_, −53.00 to −69.12 kcal/mol) and electrostatic interactions (∆*E*_elec_, −18.96 to −79.92 kcal/mol) had significant positive contributions to the final binding energy (∆*E*_bind_). Electrostatic and van der Waals interactions are often used as indicators for the evaluation of relative binding strengths [26]. In contrast, the contributions of nonpolar solvation energy (∆*G*_nonpolar_, −4.71 to −7.19 kcal/mol) were relatively small, and polar solvation energy provided a negative contribution (∆*G*_polar_, 49.23 to 111.05 kcal/mol). The findings suggested that the primary factors driving the formation of the complexes between PPARγ and four compounds were ∆*E*_vdW_ and ∆*E*_elec_.

It is noteworthy that MM-PBSA calculations yielded significantly more negative binding energies, indicating greater binding strengths than virtual screening methods. This was attributed to the fact that the Vina scoring did not utilize atomic charges for modeling electrostatic interactions [21]. Therefore, there may be a problem simulating strong electrostatic interactions between the charged parts in the Vina score. The MM-PBSA analysis provided a solution, which has been validated in multiple calculations and experiments [25]. Therefore, the employment of MD simulation and MM-PBSA significantly improve the reliability of the outcomes, rendering them necessary processes in computational research.

### 2.4. The Receptor and Ligand Interactions Analysis

**Figure 3:**
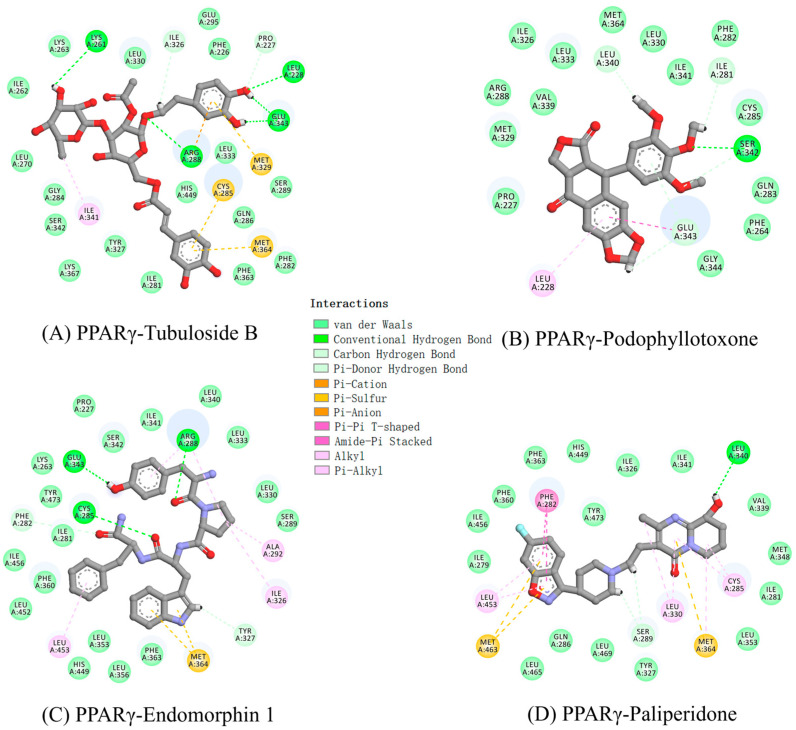
Two-dimensional diagrams of receptor−ligand interactions for PPARγ complexes with (**A**) tubuloside b; (**B**) podophyllotoxone; (**C**) endomorphin 1; (**D**) paliperidone. The figures were generated with Biovia Discovery Studio Visualizer, and the complexes are averaged structures clustered from the last 50 ns simulations.

We further explored the interaction mechanism between PPARγ and four compounds. We clustered the last 50 ns simulation trajectories and converted them into the representative binding poses for these complexes. The three-dimensional diagrams for the representative binding poses are shown in Figure S1. Biovia Discovery Studio Visualizer was used to generated two-dimensional figures to illustrate the receptor–ligand interactions (Figure 3). The complexation was primarily attributed to a diverse array of interaction types, encompassing van der Waals (vdW) contacts, hydrogen bonds, electrostatic attractions and a range of benzene-mediated interactions, specifically π-sulfur, π-alkyl and π-π stacking. Protein–ligand contact graphs are plotted for the four systems during the simulations in Figures S5–S8. Additionally, the hydrogen bond interactions played a significant role in the formation of the complex.

The analysis of hydrogen bonds serves as a crucial parameter in the detailed examination of protein–ligand complexes throughout MD simulations, providing insights into their stability and interactions. The formation of hydrogen bonds between PPARγ and specific amino acid residues has been identified as a crucial determinant in some studies [7,27]. We analyzed the number of hydrogen bond formations, as shown in Figure S9; the result found that the average number of hydrogen bonds of tubuloside b, podophyllotoxone, endomorphin 1 and paliperidone were 5, 2, 3 and 1, respectively. The results were basically consistent with the two-dimensional results (Figure 3).

In detail, one ether group in tubuloside b formed a hydrogen bond with Arg288, with a bond length of 2.2 Å. Arg288 is also a key residue located in helix H3 in PPARγ, as exhibited in partial agonists AL29-26 [28] and halofenicacid (S)-2 [29]. Two hydroxyl groups in tubuloside b formed hydrogen bonds with Leu228 and Lys261. In particular, Glu343, located in the β-sheet, formed two strong hydrogen bonds with two hydroxyl groups in tubuloside b with bond lengths of 1.47 and 1.59 Å. For podophyllotoxone, one ether group formed a hydrogen bond with a bond length of 1.88 Å with the key residue of Ser342. The role of Ser342 is an important criterion for identifying partial agonist. The binding of Ser342 with PPARγ partial agonists leads to a decrease in the stabilization of helix H12 and an increase in the stabilization of helix H3, thus affecting the recruitment of coactivators and reducing the transactivation activity of PPARγ [10,30]. One carbonyl group in endomorphin 1 exhibits a notable hydrogen bond interaction with the crucial residue of Cys285 with a bond length of 2.01 Å. The residue of Cys285 plays an important role in enhancing interactions between PPARγ and partial agonists MEKT75 [31]. Furthermore, it also formed two hydrogen bonds with Leu343 and Arg288 with bond lengths of 1.73 and 1.78 Å, respectively. The hydroxyl group of paliperidone formed a hydrogen bond with Leu340 with a bond length of 2.86 Å. It was noteworthy that the abovementioned amino acids were not the amino acids that define the compound as a full agonist rosiglitazone (His323, His449 and Tyr473) [32], suggesting the roles of compounds as potential partial agonists of PPARγ.

Following the analysis of hydrogen bonds, we examined the detailed energy contributions of each residue within the ligand-bound systems, as clearly depicted in Figures S10–S13. It was noteworthy that the residues exhibited distinct energetic behaviors, contributing favorably (negative energy values) or unfavorably (positive energy values) upon interactions with the ligands and PPARγ. This decomposition of energy contributions provided valuable insights into the underlying molecular mechanisms that govern the interactions, facilitating a deeper understanding of the system.

Within the relatively large ligand-binding domain, residues tended to congregate predominantly around structural features such as helices H2, H3, H5 and H7 and beta sheets, where partial agonists were known to bind [10]. Notably, sulfur-containing residues, including Cys285, Met329 and Met364, exhibited particularly strong negative binding energies, indicating their crucial involvement in ligand stabilization. Further non-polar residues like Phe282, Leu330, Ile326 and Phe363 appeared to have a high energy contribution to tubuloside b, endomorphin 1 and paliperidone. The favorable binding energies exhibited by podophyllotoxone were primarily contributions from the non-polar residues of Pro227, Leu228, Leu330, Ile326, Leu333 and Ile341. This specific interaction pattern distinguished the binding mechanism of podophyllotoxone from that observed in the other three systems, highlighting a unique molecular recognition process.

By far, most partial agonists do not occupy branch I of the ligand-binding pocket, thereby lacking any contact with AF2 residues. Instead, most partial agonists predominantly occupy branches II and III portions of the ligand-binding pocket [15]. Based on the analyses of binding poses and interacting residues, tubuloside b, podophyllotoxone, endomorphin 1 and paliperidone were mainly positioned in the branch II and III portions of the ligand-binding pocket, suggesting they may function as potential partial agonists.

### 2.5. TR-FRET Competitive Binding Assay and PPARγ Transactivation Assay

**Figure 4:**
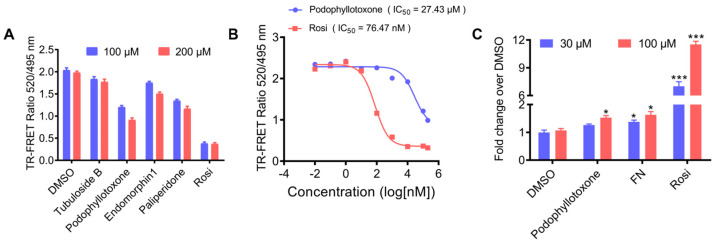
Identification of podophyllotoxone as a novel PPARγ ligand with potent binding affinity. (**A**) LanthaScreen TR-FRET measurements for the selected hits (100 μM and 200 μM) binding to PPARγ after incubating for 6 h. (**B**) Dose–response competition curves for podophyllotoxone (100 μM–500 μM) incubated for 6 h. Concentrations were expressed as a log 10 scale. (**C**) Cell-based transcription assays were used to compare the agonistic activity of podophyllotoxone and FN toward PPARγ at the concentration of 30 and 100 μM. * *p* < 0.05, *** *p* < 0.001 compared with DMSO group. (n = 3, error bar = SEM).

Encouraged by the above results, we subsequently performed a TR-FRET (Time-Resolved Fluorescence Resonance Energy Transfer) competitive binding assay to detect the binding affinities of hit compounds with PPARγ at the concentrations of 100 µM and 200 µM. DMSO and PPARγ full agonist Rosi were used as negative and positive controls, respectively. As shown in Figure 4A, podophyllotoxone revealed the highest binding affinity among the four potent hits. We further evaluated the concentration of compounds that produce 50% displacement of the tracer (IC_50_) and inhibition constant (*k_i_*) values of podophyllotoxone. As displayed in Figure 4B, the IC_50_ and *k_i_* values of podophyllotoxone were 27.43 µM and 9.86 µM, respectively. We further conducted cell-based transcription assays to evaluate the agonistic activity of podophyllotoxone toward PPARγ at concentrations of 30 µM and 100 µM. As shown in Figure 4C, Rosi displayed powerful agonistic activity against PPARγ; in contrast, podophyllotoxone exhibited weak PPARγ agonistic activity, similar to fenticonazole (FN), a PPARγ partial agonist reported in our previous study [33]. Taken together, the combined computational studies and in vitro evaluations including TR-FRET competitive binding assay and cell-based transcription assay identified podophyllotoxone as a ligand directly bound to PPARγ with partial agonistic activity.

## 3. Materials and Methods

### 3.1. Receptor and Ligand Preparations

The crystal structure of PPARγ (PDB code: 8DK4) was obtained from the RCSB Protein Data Bank database [34]. All ligand and solvent molecules in the model were removed from receptors using PyMOL (version 2.5). The missing atoms of the PPARγ protein were fixed using pdbfixer tool. The structures of compounds were collected from the PubChem compound database. The AutoDockTools module in the MGLTools package (version: 1.5.7) was used to generate the PDBQT file via adding polar hydrogens, computing Gasteiger charges and assigning AD4 atom types.

### 3.2. Docking Validation, Virtual Screening and Molecular Docking

A re-docking experiment docking the original ligand VSP-51-2 into the active site of PPARγ (PDB code: 8DK4) was conducted to evaluate the reliability of the docking procedure using the Autodock Vina software (version 1.2.3) [21]. Both the protein and the co-crystal ligand structure were processed with the procedure described above. The grid parameter file was set as the grid center X: −23.349, Y: −20.461, Z: 10.481 (dimensional units, Å) and dimensions 30 × 30 × 30 Å.

With this protocol, the crystal structure of PPARγ (PDB code: 8DK4) was adopted as receptor for the virtual screening of Targetmol L6000 Natural Product Library using the Autodock Vina software. Candidate compounds were considered for further examination in molecular dynamic simulations if they exhibit (a) favorable energy scores, (b) a high number of hydrogen bonds formed with PPARγ key residues and (c) the ability to bind to residues other than those involved in classical agonism, as defined by residues shaping the activation function surface 2 (His323, Tyr473 and His449).

### 3.3. Molecular Dynamics Simulation

To gain insights into the relative dynamics and behavioral changes of PPARγ upon interactions with different ligands, we conducted comprehensive molecular dynamic simulations encompassing five distinct systems: apo PPARγ, as well as the tubuloside b-, podophyllotoxone-, endomorphin 1- and paliperidone-bound PPARγ complexes. Molecular dynamics simulations were carried with GROMACS (version 2021.6) [35]. We optimized eight ligands at B97-3c in the water with ORCA (version 5.0.4) [36] and then calculated the restrained electrostatic potential (RESP) charges with the aid of Multiwfn (version 3.8) [37]. The Amber99SB-ILDN force field [38] was employed to model PPARγ and ions, and the general Amber force field (GAFF) [39] was chosen for the ligands. The rigid SPC model [40] was used to model water molecules. The protein–ligand complex was immersed in a cubic box and the minimum value between the solvent and the nearest box edge was 1.0 nm. The system was neutralized by adding a corresponding number of sodium or chloride (Na^+^/Cl^−^) ions. We performed an energy minimization step on the system using the steepest descent algorithm to simply remove any unreasonable contacts between atoms. For the equilibration phase, a short NPT equilibration was carried out for 0.5 ns. The system was equilibrated with a temperature coupling (298.15 K) using a V-rescale thermostat [41] and a pressure coupling using the C-rescale coupling algorithm [42]. Finally, 200 ns production simulations were performed under the NPT ensemble with the time step set as 2 fs. The simulated temperature was set to 298.15 K while the pressure was set to 1 bar. The Particle mesh Ewald (PME) [43] method was employed to treat the long-range electrostatic interactions and the cut off of van der Waals interaction was set to 1.2 nm. The trajectories were analyzed by tools in the GROMACS suite, including Root Mean Square Deviation (RMSD), Root Mean Square Fluctuation (RMSF), Radius of Gyration (RG) and Solvent-Accessible Surface Area (SASA).

### 3.4. Calculation of the Binding Energy

The molecular mechanics Poisson–Boltzmann surface area (MM-PBSA) is a popular method to estimate the binding energy between the receptor and ligand. It is more accurate than most fast scoring approaches in the docking and has a relatively lower computation load than the free energy calculations with an explicit solvent. 

After 150 ns MD simulations, we stripped water molecules and ions from the systems, and extracted 50 conformations of complexes from the last 50 ns trajectory with an interval of 1000 ps. Decomposition of the binding energies (kcal/mol) of the ligands with PPARγ using the MM-PBSA analysis of the last 50 ns simulation trajectories. The gmx_MMPBSA toolkit [44] was used to compute the binding energy (∆*E*_bind_) that includes the contributions from van der Waals and electrostatic interactions as well as the polar (∆*G*_polar_) and nonpolar (∆*G*_nonpolar_) solvation energies. Together with an entropy contribution (−*T*∆*S*), one can obtain the binding free energy (∆*G*_bind_), as provided in Equation (1):

∆*G*_bind_ = ∆*E*_MM_ + ∆*G*_sol_ − *T*∆*S* = ∆*E*_vdW_ + ∆*E*_elec_ + ∆*G*_polar_ + ∆*G*_nonpolar_ − *T*∆*S* (1)

where ∆*E*_MM_ denotes vacuum potential energy and is the sum of van der Waals (∆*E*_vdW_) and electrostatic (∆*E*_elec_) contributions. ∆*G*_sol_ can be decomposed into polar (∆*G*_polar_) and nonpolar (∆*G*_nonpolar_) solvation contributions. *T*∆*S* refers to the conformational entropy contribution at temperature *T.* The entropy calculations typically dominate the computational cost of the MM-PBSA estimates. Due to the large computational cost, this term is neglected in most cases of practical applications [44]. Therefore, the binding energy (∆*E*_bind_) was used for comparing different ligands against PPARγ.

### 3.5. Materials

The rosiglitazone (Rosi), fenticonazole (FN), tubuloside b, podophyllotoxone, endomorphin 1 and paliperidone were purchased from MedChem Express (Monmouth Junction, NJ, USA). The LanthaScreen™ TR-FRET PPARγ competitive binding assay kit (PV4894) and Lipofectamin 2000 was obtained from Invitrogen (Carlsbad, CA, USA). PPRE × 3 TK-luciferase plasmid, renilla luciferase plasmid and hPPARγ plasmid were constructed by Obio Technology (Shanghai, China). Dual luciferase reporter assay kits (Promega, Madison, WI, USA) were also utilized.

### 3.6. TR-FRET Competitive Binding Assay

The LanthaScreen™ TR-FRET PPARγ competitive binding assay kit (PV4894) was utilized to assess the binding affinities of tubuloside b, podophyllotoxone, endomorphin 1 and paliperidone toward PPARγ. Following the manufacturer’s detailed instructions, 20 μL of each test compound, 10 μL of Fluormone™ pan-PPARγ Green and 10 μL of PPARγ LBD/Tb anti-GST Ab were added into a 384-well microtiter plate and made the final concentrations of Fluormone™ pan-PPARγ Green, PPARγ LBD and Tb anti-GST Ab 5 nM, 5 nM and 0.5 nM, respectively. Subsequently, the plate was gently mixed on an orbital plate shaker for 30 s and then incubated in the dark for 6 h at room temperature (20–25 °C). In this experimental setup, DMSO served as the negative control group, while Rosi (a known PPARγ full agonist) was used as the positive control group, allowing for the accurate evaluation of the test compounds’ binding affinities relative to these standards. Subsequently, the fluorescent emission signals from each well were measured at both 495 nm and 520 nm utilizing a multi-mode reader. The TR-FRET ratio was calculated by dividing the emission signal obtained at 520 nm and 495 nm; the decreased ratio of compounds indicates their potent binding affinity. A competition curve was then generated by plotting this TR-FRET ratio against the logarithmic concentration of the test compounds. The more potent binding affinity of the compound towards PPARγ was reflected by a decrease in the TR-FRET ratio, providing a quantitative measure of their interaction strength with the receptor. The inhibition constant (*K_i_*) for competitor was calculated by applying the Cheng–Prusoff equation as given in Equation (2):

K_i_ = IC_50_/(1 + [tracer]/K_d_) (2)

where IC_50_ is the concentration of the competitor that produces 50% displacement of the tracer, [tracer] is the concentration of Fluormone™ pan-PPAR Green used in the assay (5 nM) and K_d_ is the binding constant of Fluormone™ pan-PPAR Green to PPARγ-LBD.

### 3.7. PPARγ Transactivation Assay

Based on our preceding studies [27,33,45], cos-7 cells were transfected with hPPARγ, PPRE × 3 TK-luciferase plasmids and renilla luciferase plasmids by using Lipofectamin 2000. After 24 h of incubation, the transfected cells were treated with DMSO, podophyllotoxone, FN and Rosi at a concentration of 30 or 100 μM for another 24 h. Ultimately, dual luciferase reporter assay kits were used to detect luciferase activities. Briefly, (1) 1 × passive lysis buffer was added into 24 well plates to lyse cells for 15 min; (2) 20 μL cell lysate and 100 μL luciferase assay reagent II were mixed into the 96-well plates, and the fluorescence values were immediately detected with a multifunction microplate reader (Bioteck, Synegy1), which represented the luciferase activity; (3) 100 μL Stop & Glo^®^ Reagent was added into the above mixture and the fluorescence values were detected, which represented the renilla activity. The luciferase activity was normalized to renilla activity. Each experiment was repeated three times, with DMSO serving as the negative control and rosiglitazone (Rosi) serving as the positive control.

## 4. Discussion

In recent years, synthetic PPARγ partial agonists [27,33,45,46] have emerged as a promising alternative to full agonists, demonstrating hypoglycemic efficacy while mitigating the risk of severe side effects commonly associated with full agonists, including weight gain, fluid retention and heart failure. Despite the promising potential of PPARγ-targeting agents for treating T2DM, no such anti-diabetic agents specifically designed to modulate PPARγ have yet been translated into clinical practice. Upon conducting virtual screening of the Targetmol L6000 Natural Product Library, utilizing both molecular dynamics simulations and molecular mechanics Poisson–Boltzmann surface area (MM-PBSA) approaches, we have identified four compounds: tubuloside b, podophyllotoxone, endomorphin 1 and paliperidone, as promising candidates for PPARγ partial agonists.

In MM-PBSA calculations, tubuloside b showed the highest binding energy for PPARγ, followed by endomorphin 1, podophyllotoxone and paliperidone. We further utilized a TR-FRET competitive binding assay experiment to find out the binding affinity between these ligands and PPARγ. To our surprise, the binding affinity results were quite different from the MM-PBSA calculation results. Tubuloside b, endomorphin 1 and paliperidone showed low binding affinity with PPARγ, while podophyllotoxone emerged as the most potent compound among the four identified hits. Cell-based transcription assays further showed that podophyllotoxone exhibits partial agonistic activity.

The differences of binding affinities observed between molecular simulations and in vitro experiments were mainly attributed to the distinct chemical structures and orientations of the chemical groups in the compounds. In molecular dynamics simulations, such inconsistencies are often encountered and can be attributed to various factors, including the inherent limitations of the computational models and the complexity of the underlying biological systems. From a systematic evaluation of the prediction capabilities of MM-PBSA methods, researchers have concluded that the accuracy of binding free energy predictions is related to several crucial factors, including force field, charge model, continuum solvation method, interior dielectric constant and sampling method [47]. Nevertheless, computational simulations continue to play a pivotal role in drug discovery.

## 5. Conclusions

In this work, virtual screening of Targetmol L6000 Natural Product Library leaded to the discovery of tubuloside b, podophyllotoxone, endomorphin 1 and paliperidone as potential PPARγ partial agonists. The subsequent evaluation of RMSD, RMSF, RG and SASA values of complexes between four compounds and PPARγ LBD by performing molecular dynamic simulations indicated that the four complexes were stable. The MM-PBSA calculations revealed the binding energies of four compounds ranged from −28.08 to −45.18 kcal/mol, suggesting their potent binding affinities with PPARγ. Further in vitro evaluations including TR-FRET competitive binding assays and cell-based transcription assays demonstrated podophyllotoxone functions as a PPARγ partial agonist. Our research has yielded an effective strategy for the identification of novel PPARγ partial agonists. Additionally, we have identified a scaffold that can be utilized for structural optimization, with the goal of enhancing binding affinity while preserving the partial agonistic activity of the agonists.

## Figures and Tables

**Table 1:** Top four compounds from virtual screening of the Targetmol L6000 Natural Product Library via Autodock Vina.

Name	CAS	Molecular Structure	Docking Score (kcal/mol)	Hydrogen Bond	Hydrophobic Interaction
Tubuloside B	112516-04-8	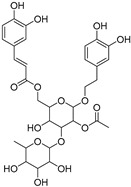	−10.07	Leu228, Gln286, Arg288, Ser342, Glu343	Phe282, Arg288, Ala292, Met329, Leu333
Podophyllotoxone	477-49-6	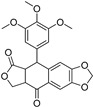	−10.04	Ser342	Arg288, Ala292, Ile326, Leu330, Ile341
Endomorphin 1	189388-22-5	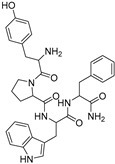	−10.68	Gly284, Cys285 Arg288	Leu228, Met329, Leu333, Arg288, Phe287, Lys263, Ile341, Cys285, Met364, Val339, Met348, Leu353, Ile326
Paliperidone	144598-75-4	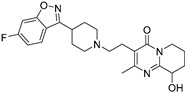	−10.44	Ser342	Leu228, Leu333, Ile341, Arg288, Ala292, Ile326, Leu330, Cys285, His449, Phe363, Tyr327

**Table 2:** Calculated binding energies (kcal/mol) of the ligands with PPARγ by MM-PBSA using MD-derived trajectories as well as the energy decomposition.

Compound	∆*E*_vdW_	∆*E*_elec_	∆*E*_MM_	∆*G*_polar_	∆*G*_nonpolar_	∆*G*_sol_	∆*E*_bind_
Tubuloside B	−69.12 ± 0.43	−79.92 ± 1.61	−149.04 ± 1.62	111.05 ± 1.25	−7.19 ± 0.02	103.86 ± 1.24	−45.18 ± 0.70
Podophyllotoxone	−53.00 ± 0.38	−21.67 ± 0.49	−74.67 ± 0.61	49.23 ± 0.41	−4.71 ± 0.01	44.52 ± 0.41	−30.15 ± 0.38
Endomorphin 1	−66.03 ± 0.38	−54.33 ± 1.39	−120.35 ± 1.49	88.34 ± 1.26	−6.58 ± 0.02	81.49 ± 1.25	−38.86 ± 0.54
Paliperidone	−55.47 ± 0.29	−18.96 ± 0.53	−74.43 ± 0.60	51.56 ± 0.53	−5.21 ± 0.01	46.35 ± 0.52	−28.08 ± 0.34

∆*E*_MM_ is the MM part and amounts to ∆*E*_vdW_ + ∆*E*_elec_. ∆*G*_sol_ is the solvation energies (∆*G*_polar_ + ∆*G*_nonpolar_). For each compound, 50 frames from the last 50 ns trajectories were used for the MM-PBSA analysis.

## Data Availability

Data is contained within the article and Supplementary Materials.

## Supplementary Materials

The following supporting information can be downloaded at: https://www.mdpi.com/article/10.3390/molecules29204881/s1, Table S1: Average value of RMSD, RMSF, RG and SASA; Figure S1: Three-dimensional diagrams of PPARγ protein with (A) tubuloside b; (B) podophyllotoxone; (C) endomorphin 1; (D) paliperidone; Figure S2: Analysis of RMSF of tubuloside b-, podophyllotoxone-, endomorphin 1-, paliperidone-bound and apo PPARγ; Figure S3: Analysis of RG of tubuloside b-, podophyllotoxone-, endomorphin 1-, paliperidone-bound and apo PPARγ; Figure S4: Analysis of SASA of tubuloside b-, podophyllotoxone-, endomorphin 1-, paliperidone-bound and apo PPARγ; Figure S5: Protein–ligand contacts between PPARγ and tubuloside b; H-bonds are represented as green color, purple-colored bars are depicted for hydrophobic interactions, ionic bonds are shown in blue color; Figure S6: Protein–ligand contacts between PPARγ and podophyllotoxone. H-bonds are represented as green color, purple-colored bars are depicted for hydrophobic interactions, ionic bonds are shown in blue color; Figure S7: Protein–ligand contacts between PPARγ and endomorphin 1. H-bonds are represented as green color, purple-colored bars are depicted for hydrophobic interactions, ionic bonds are shown in blue color; Figure S8: Protein–ligand contacts between PPARγ and paliperidone. H-bonds are represented as green color, purple-colored bars are depicted for hydrophobic interactions; Figure S9: Number of hydrogen bonds of protein–ligand interaction for the complexes; Figure S10: Energy contribution per residue to the binding of PPARγ with tubuloside b; Figure S11: Energy contribution per residue to the binding of PPARγ with podophyllotoxone; Figure S12: Energy contribution per residue to the binding of PPARγ with endomorphin 1; Figure S13: Energy contribution per residue to the binding of PPARγ with paliperidone.

## References

[1] Ong K.L., Stafford L.K., McLaughlin S.A., Boyko E.J., Vollset S.E., Smith A.E., Dalton B.E., Duprey J., Cruz J.A., Hagins H. (2023). Global, regional, and national burden of diabetes from 1990 to 2021, with projections of prevalence to 2050: A systematic analysis for the Global Burden of Disease Study 2021. Lancet.

[2] Demir S., Nawroth P.P., Herzig S., Ekim Üstünel B. (2021). Emerging Targets in Type 2 Diabetes and Diabetic Complications. Adv. Sci..

[3] Rangwala S.M., Lazar M.A. (2004). Peroxisome proliferator-activated receptor γ in diabetes and metabolism. Trends Pharmacol. Sci..

[4] Janani C., Ranjitha Kumari B.D. (2015). PPAR gamma gene—A review. Diabetes Metab. Syndr. Clin. Res. Rev..

[5] Chigurupati S., Dhanaraj S.A., Balakumar P. (2015). A step ahead of PPARγ full agonists to PPARγ partial agonists: Therapeutic perspectives in the management of diabetic insulin resistance. Eur. J. Pharmacol..

[6] Ahmadian M., Suh J.M., Hah N., Liddle C., Atkins A.R., Downes M., Evans R.M. (2013). PPARγ signaling and metabolism: The good, the bad and the future. Nat. Med..

[7] Kroker A.J., Bruning J.B. (2015). Review of the Structural and Dynamic Mechanisms of PPARγ Partial Agonism. PPAR Res..

[8] Chan L.S.A., Wells R.A., Shi X.-M. (2009). Cross-Talk between PPARs and the Partners of RXR: A Molecular Perspective. PPAR Res..

[9] Lebovitz H.E. (2019). Thiazolidinediones: The Forgotten Diabetes Medications. Curr. Diabetes Rep..

[10] Choi J.H., Banks A.S., Estall J.L., Kajimura S., Boström P., Laznik D., Ruas J.L., Chalmers M.J., Kamenecka T.M., Blüher M. (2010). Anti-diabetic drugs inhibit obesity-linked phosphorylation of PPARγ by Cdk5. Nature.

[11] Rennings A.J.M., Smits P., Stewart M.W., Tack C.J. (2006). Fluid Retention and Vascular Effects of Rosiglitazone in Obese, Insulin-Resistant, Nondiabetic Subjects. Diabetes Care.

[12] Guan Y., Hao C., Cha D.R., Rao R., Lu W., Kohan D.E., Magnuson M.A., Redha R., Zhang Y., Breyer M.D. (2005). Thiazolidinediones expand body fluid volume through PPARγ stimulation of ENaC-mediated renal salt absorption. Nat. Med..

[13] Rosen C.J. (2010). Revisiting the Rosiglitazone Story—Lessons Learned. N. Engl. J. Med..

[14] Weidner C., de Groot J.C., Prasad A., Freiwald A., Quedenau C., Kliem M., Witzke A., Kodelja V., Han C.-T., Giegold S. (2012). Amorfrutins are potent antidiabetic dietary natural products. Proc. Natl. Acad. Sci. USA.

[15] Bruning J.B., Chalmers M.J., Prasad S., Busby S.A., Kamenecka T.M., He Y., Nettles K.W., Griffin P.R. (2007). Partial Agonists Activate PPARγ Using a Helix 12 Independent Mechanism. Structure.

[16] Frkic R.L., Pederick J.L., Horsfall A.J., Jovcevski B., Crame E.E., Kowalczyk W., Pukala T.L., Chang M.R., Zheng J., Blayo A.-L. (2023). PPARγ Corepression Involves Alternate Ligand Conformation and Inflation of H12 Ensembles. ACS Chem. Biol..

[17] Frkic R.L., Marshall A.C., Blayo A.-L., Pukala T.L., Kamenecka T.M., Griffin P.R., Bruning J.B. (2018). PPARγ in Complex with an Antagonist and Inverse Agonist: A Tumble and Trap Mechanism of the Activation Helix. iScience.

[18] Chen F., Ma L., Cai G., Tang J., Wang Y., Liu Q., Liu X., Hou N., Zhou Z., Yi W. (2022). Identification of a novel PPARγ modulator with good anti-diabetic therapeutic index via structure-based screening, optimization and biological validation. Biomed. Pharmacother..

[19] Omoboyowa D.A., Singh G., Fatoki J.O., Oyeneyin O.E. (2022). Computational investigation of phytochemicals from Abrus precatorius seeds as modulators of peroxisome proliferator-activated receptor gamma (PPARγ). J. Biomol. Struct. Dyn..

[20] Chu Y., Gui S., Zheng Y., Zhao J., Zhao Y., Li Y., Chen X. (2024). The natural compounds, Magnolol or Honokiol, promote adipose tissue browning and resist obesity through modulating PPARα/γ activity. Eur. J. Pharmacol..

[21] Eberhardt J., Santos-Martins D., Tillack A.F., Forli S. (2021). AutoDock Vina 1.2.0: New Docking Methods, Expanded Force Field, and Python Bindings. J. Chem. Inf. Model..

[22] Mandal S., Faizan S., Raghavendra N.M., Kumar B.R.P. (2022). Molecular dynamics articulated multilevel virtual screening protocol to discover novel dual PPAR α/γ agonists for anti-diabetic and metabolic applications. Mol. Divers..

[23] Pathak R.K., Gupta A., Shukla R., Baunthiyal M. (2018). Identification of new drug-like compounds from millets as Xanthine oxidoreductase inhibitors for treatment of Hyperuricemia: A molecular docking and simulation study. Comput. Biol. Chem..

[24] Zhou B., Zhang Y., Jiang W., Zhang H. (2022). Virtual Screening of FDA-Approved Drugs for Enhanced Binding with Mitochondrial Aldehyde Dehydrogenase. Molecules.

[25] Genheden S., Ryde U. (2015). The MM/PBSA and MM/GBSA methods to estimate ligand-binding affinities. Expert Opin. Drug Discov..

[26] Jiang W., Chen J., Zhang P., Zheng N., Ma L., Zhang Y., Zhang H. (2023). Repurposing Drugs for Inhibition against ALDH2 via a 2D/3D Ligand-Based Similarity Search and Molecular Simulation. Molecules.

[27] Ma L., Tang J., Cai G., Chen F., Liu Q., Zhou Z., Zhang S., Liu X., Hou N., Yi W. (2022). Structure-based screening and biological validation of the anti-thrombotic drug-dicoumarol as a novel and potent PPARγ-modulating ligand. Bioorganic Chem..

[28] Capelli D., Cerchia C., Montanari R., Loiodice F., Tortorella P., Laghezza A., Cervoni L., Pochetti G., Lavecchia A. (2016). Structural basis for PPAR partial or full activation revealed by a novel ligand binding mode. Sci. Rep..

[29] Laghezza A., Montanari R., Lavecchia A., Piemontese L., Pochetti G., Iacobazzi V., Infantino V., Capelli D., De Bellis M., Liantonio A. (2015). On the Metabolically Active Form of Metaglidasen: Improved Synthesis and Investigation of Its Peculiar Activity on Peroxisome Proliferator-Activated Receptors and Skeletal Muscles. ChemMedChem.

[30] de Groot J.C., Weidner C., Krausze J., Kawamoto K., Schroeder F.C., Sauer S., Büssow K. (2013). Structural Characterization of Amorfrutins Bound to the Peroxisome Proliferator-Activated Receptor γ. J. Med. Chem..

[31] Ohashi M., Gamo K., Oyama T., Miyachi H. (2015). Peroxisome proliferator-activated receptor gamma (PPARγ) has multiple binding points that accommodate ligands in various conformations: Structurally similar PPARγ partial agonists bind to PPARγ LBD in different conformations. Bioorganic Med. Chem. Lett..

[32] Ahsan W. (2019). The Journey of Thiazolidinediones as Modulators of PPARs for the Management of Diabetes: A Current Perspective. Curr. Pharm. Des..

[33] Ma L., Lian Y., Tang J., Chen F., Gao H., Zhou Z., Hou N., Yi W. (2021). Identification of the anti-fungal drug fenticonazole nitrate as a novel PPARγ-modulating ligand with good therapeutic index: Structure-based screening and biological validation. Pharmacol. Res..

[34] Burley S.K., Bhikadiya C., Bi C., Bittrich S., Chao H., Chen L., Craig P.A., Crichlow G.V., Dalenberg K., Duarte J.M. (2023). RCSB Protein Data Bank (RCSB.org): Delivery of experimentally-determined PDB structures alongside one million computed structure models of proteins from artificial intelligence/machine learning. Nucleic Acids Res..

[35] Páll S., Zhmurov A., Bauer P., Abraham M., Lundborg M., Gray A., Hess B., Lindahl E. (2020). Heterogeneous parallelization and acceleration of molecular dynamics simulations in GROMACS. J. Chem. Phys..

[36] Neese F. (2022). Software update: The ORCA program system—Version 5.0. Wiley Interdiscip. Rev. Comput. Mol. Sci..

[37] Lu T., Chen F. (2011). Multiwfn: A multifunctional wavefunction analyzer. J. Comput. Chem..

[38] Lindorff-Larsen K., Piana S., Palmo K., Maragakis P., Klepeis J.L., Dror R.O., Shaw D.E. (2010). Improved side-chain torsion potentials for the Amber ff99SB protein force field. Proteins.

[39] Wang J., Wolf R.M., Caldwell J.W., Kollman P.A., Case D.A. (2004). Development and testing of a general amber force field. J. Comput. Chem..

[40] Jorgensen W.L., Chandrasekhar J., Madura J.D., Impey R.W., Klein M.L. (1983). Comparison of simple potential functions for simulating liquid water. J. Chem. Phys..

[41] Bussi G., Donadio D., Parrinello M. (2007). Canonical sampling through velocity rescaling. J. Chem. Phys..

[42] Bernetti M., Bussi G. (2020). Pressure control using stochastic cell rescaling. J. Chem. Phys..

[43] Essmann U., Perera L., Berkowitz M.L., Darden T., Lee H., Pedersen L.G. (1995). A smooth particle mesh Ewald method. J. Chem. Phys..

[44] Valdés-Tresanco M.S., Valdés-Tresanco M.E., Valiente P.A., Moreno E. (2021). gmx_MMPBSA: A New Tool to Perform End-State Free Energy Calculations with GROMACS. J. Chem. Theory Comput..

[45] Ma L., Tang J., Chen F., Liu Q., Huang J., Liu X., Zhou Z., Yi W. (2024). Structure-based screening, optimization and biological evaluation of novel chrysin-based derivatives as selective PPARγ modulators for the treatment of T2DM and hepatic steatosis. Eur. J. Med. Chem..

[46] Jiang H., Zhou X.E., Shi J., Zhou Z., Zhao G., Zhang X., Sun Y., Suino-Powell K., Ma L., Gao H. (2020). Identification and structural insight of an effective PPARγ modulator with improved therapeutic index for anti-diabetic drug discovery. Chem. Sci..

[47] Wang E., Sun H., Wang J., Wang Z., Liu H., Zhang J.Z.H., Hou T. (2019). End-Point Binding Free Energy Calculation with MM/PBSA and MM/GBSA: Strategies and Applications in Drug Design. Chem. Rev..

