# Supplementary material for: Identification of Novel PPARγ Partial Agonists Based on Virtual Screening Strategy: In Silico and In Vitro Experimental Validation
Source: Molecules. 2024 Oct 15;29(20):4881. doi: 10.3390/molecules29204881 (PMC11509912; doi:10.3390/molecules29204881)
Supplement: Supplementary file 1 [file molecules-29-04881-s001.zip › molecules-3082179-SI.pdf]

Supplementary Materials for:

Identification of novel PPARγ partial agonists based on virtual screening strategy: in silico and in vitro experimental validation

Yu-E Lian [1,†], Mei Wang[1,†], Lei Ma[1,†], Wei Yi[1,*] , Siyan Liao[1], Hui Gao[1,*] and Zhi Zhou[1,*]

1 School of Pharmaceutical Sciences, Guangzhou Medical University, Guangzhou, Guangdong 511436, China.

* Correspondence: yiwei@gzhmu.edu.cn (W.Y.); gaoh9@gzhmu.edu.cn (H.G.); zhouzhi@gzhmu.edu.cn (Z.Z.).

† These authors contributed equally to this work.

Table S1. Average value of RMSD, RMSF, RG and SASA.

System	RMSD (nm)	RMSF (nm)	RG (nm)	SASA (nm^2)
PPARγ-Tubuloside B	0.24	0.08	1.91	147.83
PPARγ-Podophyllotoxone	0.25	0.08	1.90	146.27
PPARγ-Endomorphin 1	0.20	0.07	1.92	149.15
PPARγ-Paliperidone	0.24	0.08	1.92	146.72
PPARγ	0.28	0.09	1.58	148.54

The last 50 ns trajectories of MD simulations were used for the calculation.

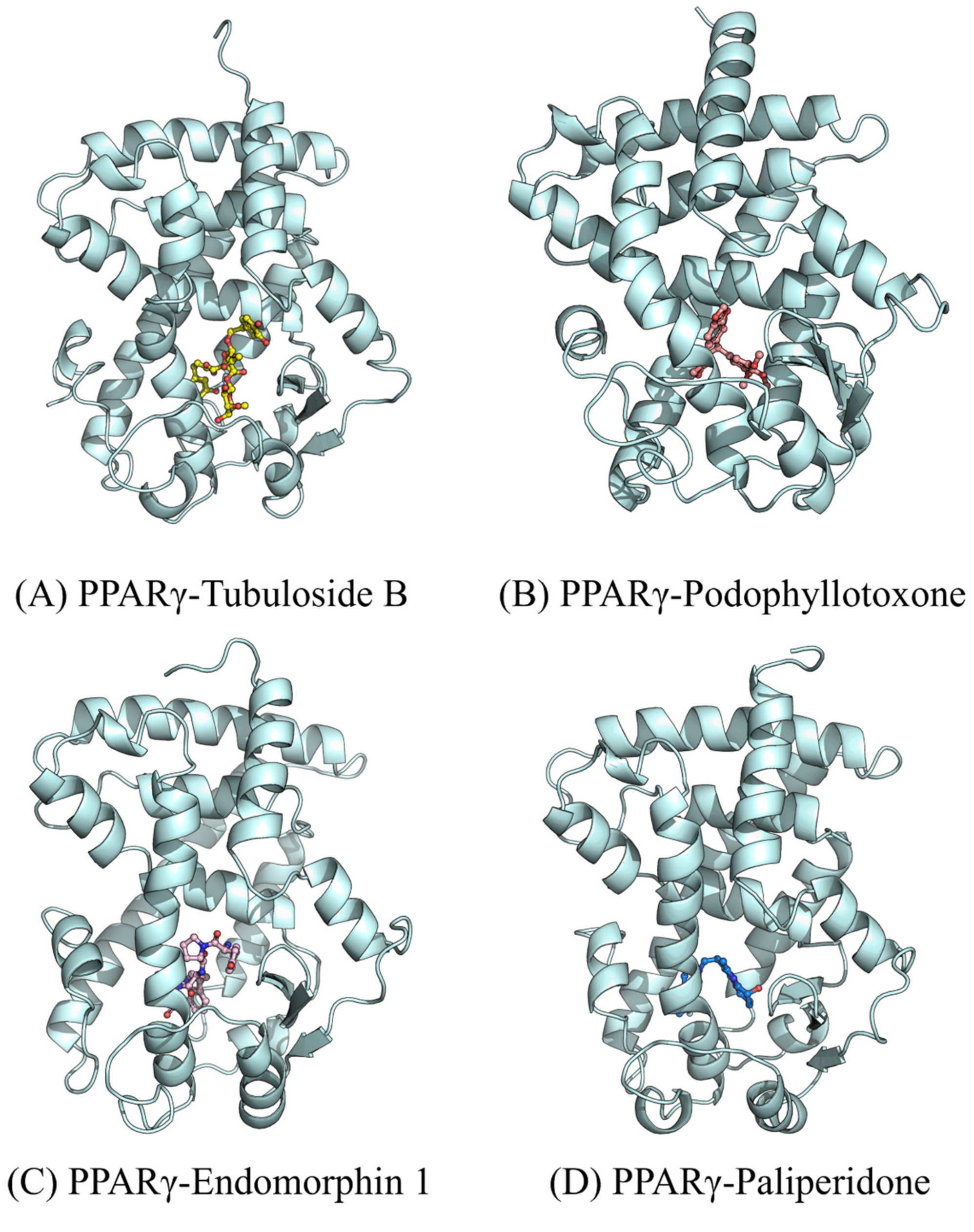

Figure S1. Three-dimensional diagrams of PPARγ protein with (A) tubuloside b; (B) podophyllotoxone; (C) endomorphin 1; (D) paliperidone.

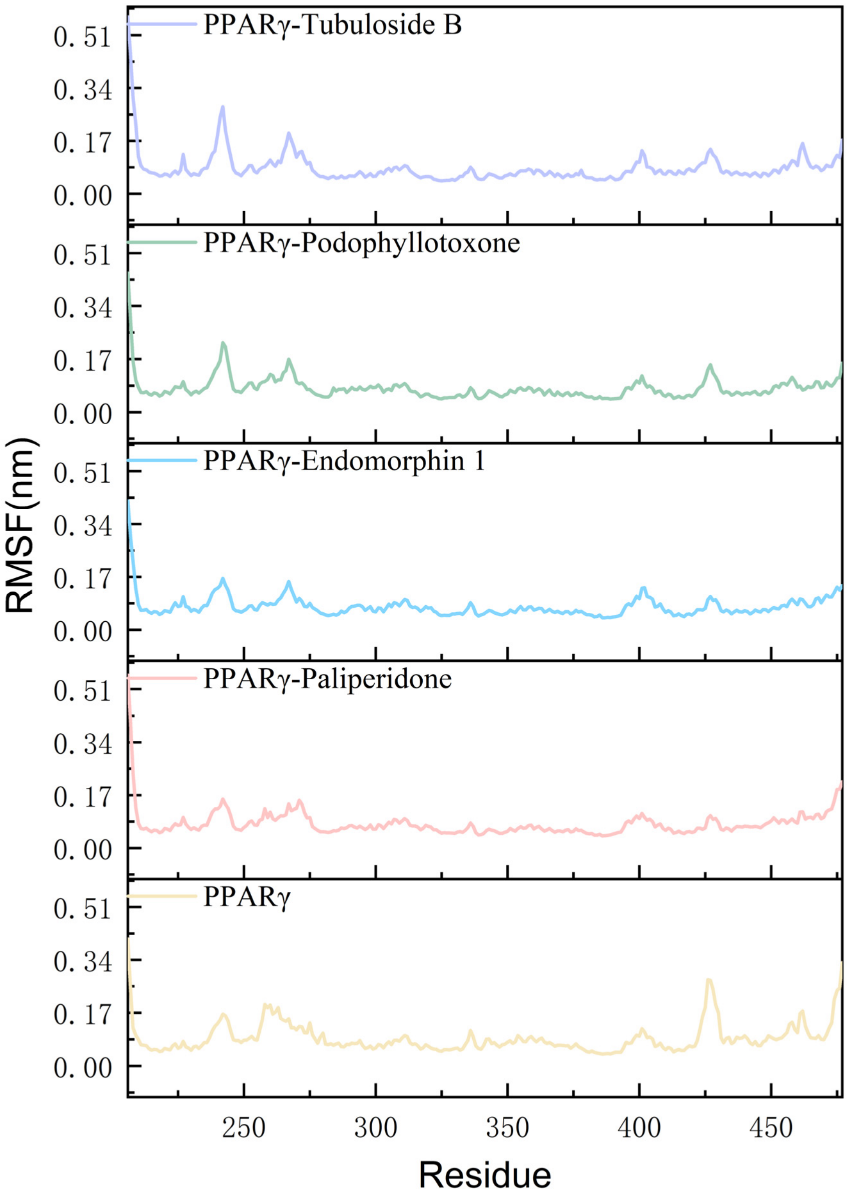

Figure S2. Analysis of RMSF of tubuloside b-, podophyllotoxone-, endomorphin 1-, paliperidone-bound and apo PPARγ.

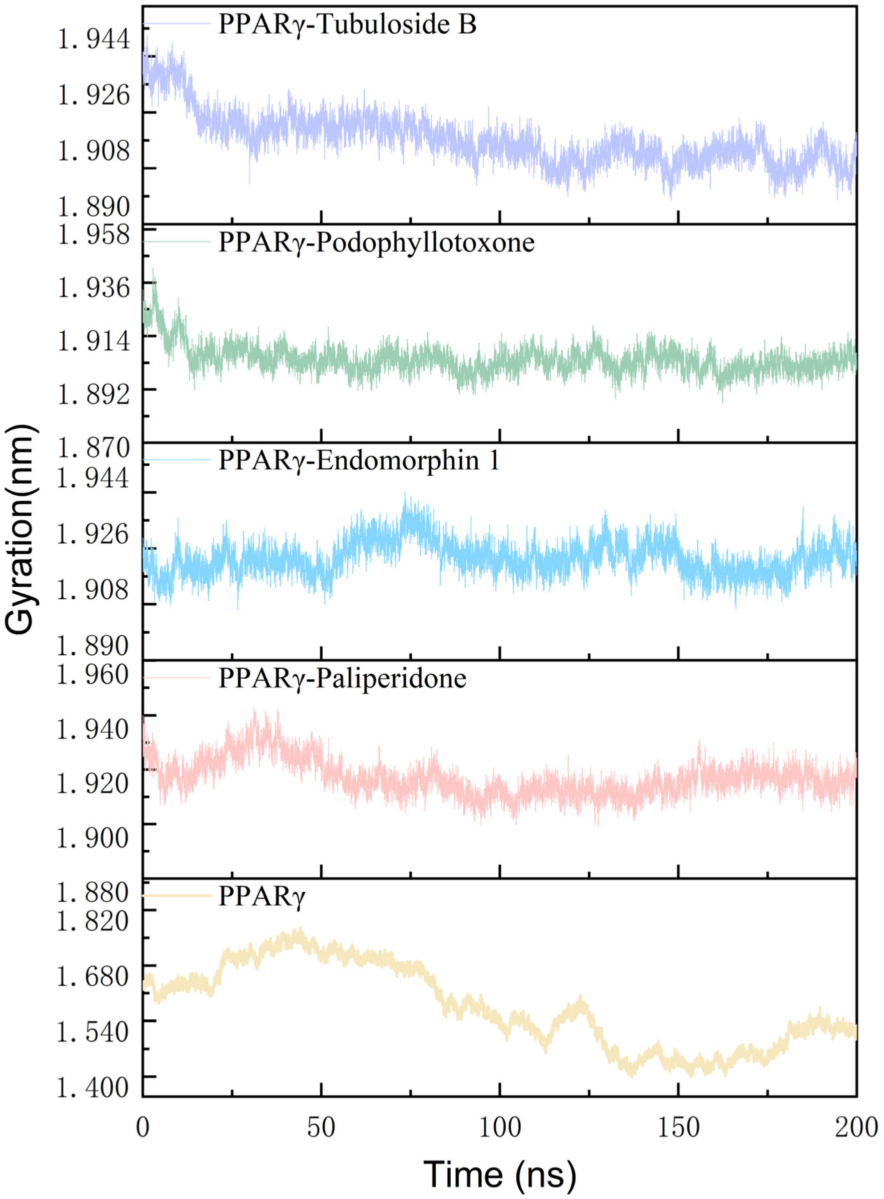

Figure S3. Analysis of RG of tubuloside b-, podophyllotoxone-, endomorphin 1-, paliperidone-bound and apo PPARγ.

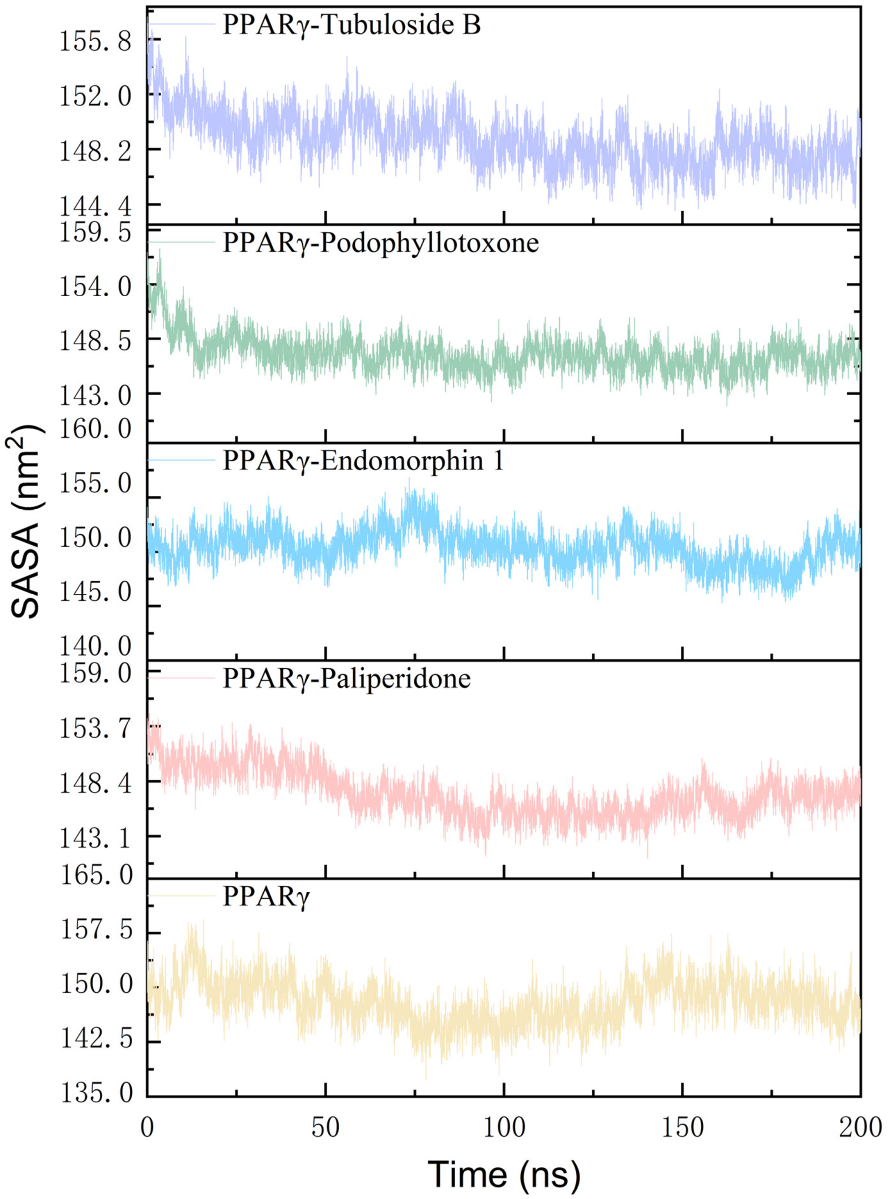

Figure S4. Analysis of SASA of tubuloside b-, podophyllotoxone-, endomorphin 1-, paliperidone-bound and apo PPARγ.

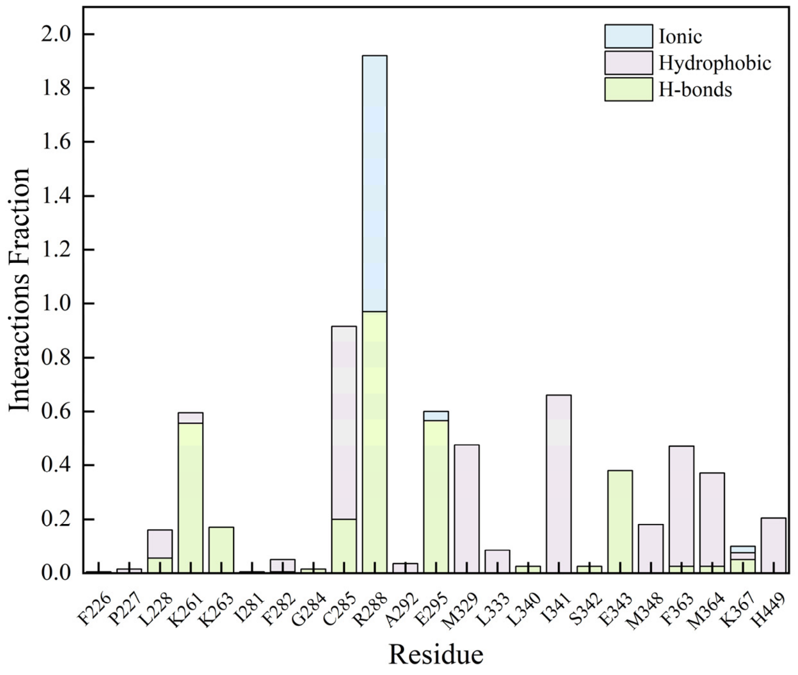

Figure S5. Protein-ligand contacts between PPARγ and tubuloside b. H-bonds are represented as green color, purple-colored bars are depicted for hydrophobic interactions, ionic bonds are shown in blue color.

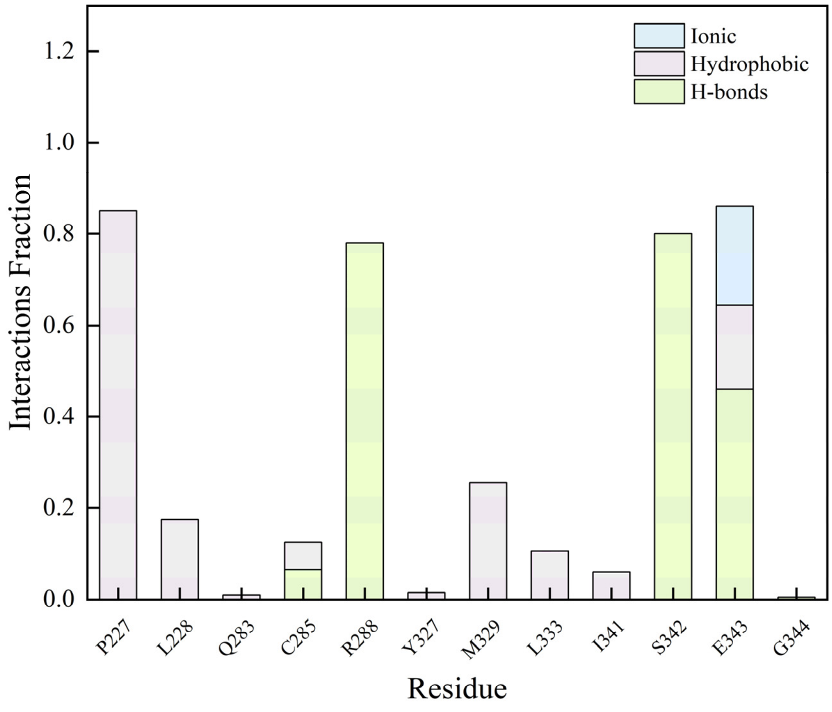

Figure S6. Protein-ligand contacts between PPARγ and podophyllotoxone. H-bonds are represented as green color, purple-colored bars are depicted for hydrophobic interactions, ionic bonds are shown in blue color.

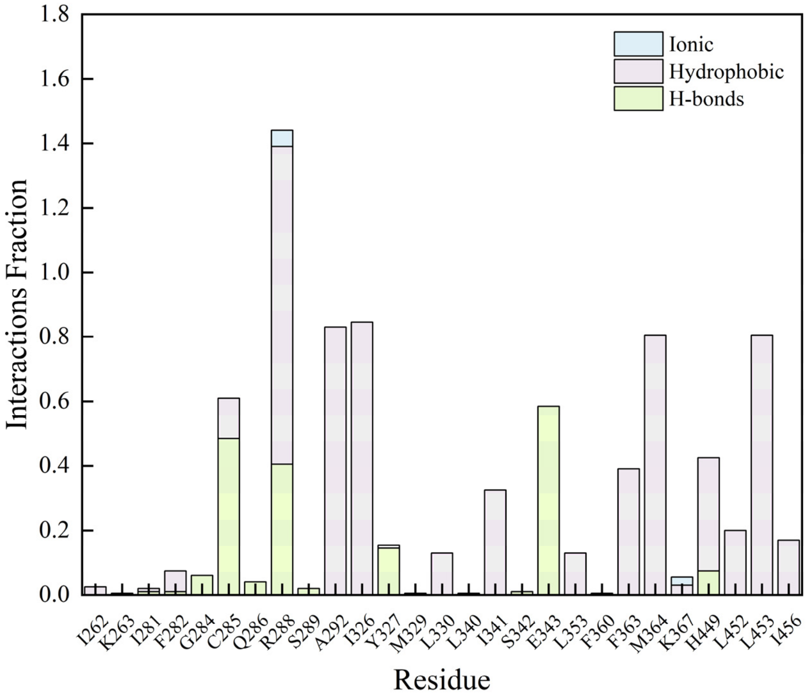

Figure S7. Protein-ligand contacts between PPARγ and endomorphin 1. H-bonds are represented as green color, purple-colored bars are depicted for hydrophobic interactions, ionic bonds are shown in blue color.

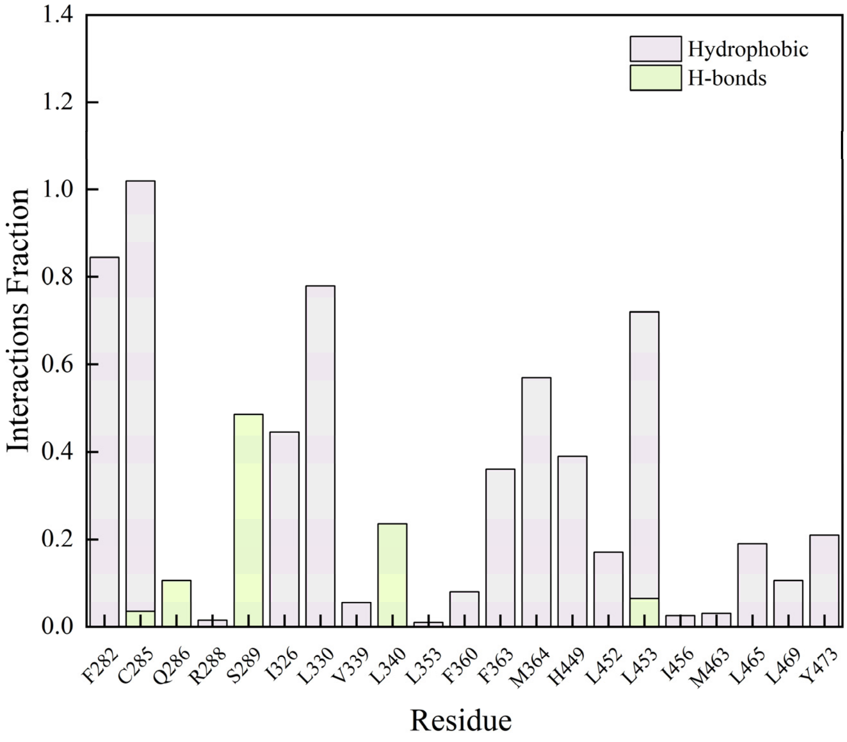

Figure S8. Protein-ligand contacts between PPARγ and paliperidone. H-bonds are represented as green color, purple-colored bars are depicted for hydrophobic interactions.

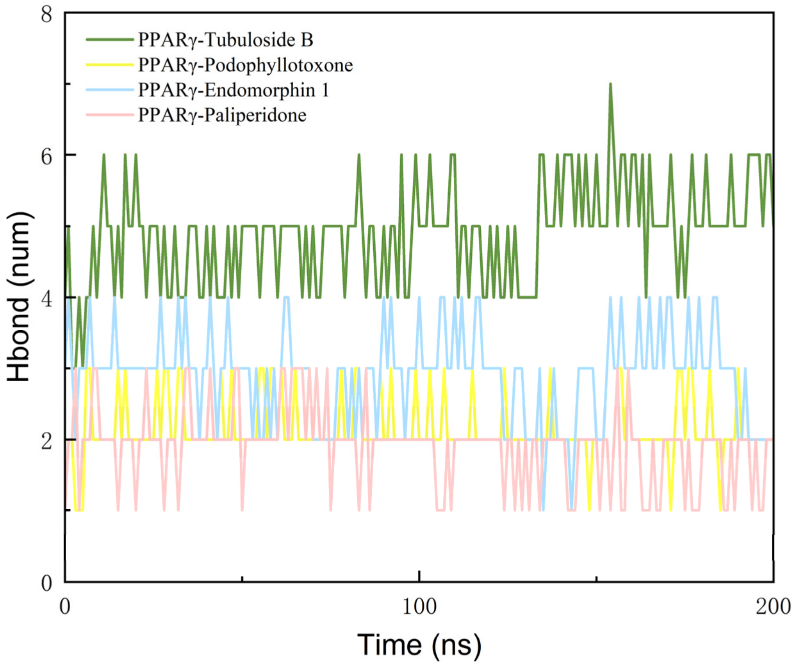

Figure S9. Number of hydrogen bonds of protein-ligand interaction for the complexes.

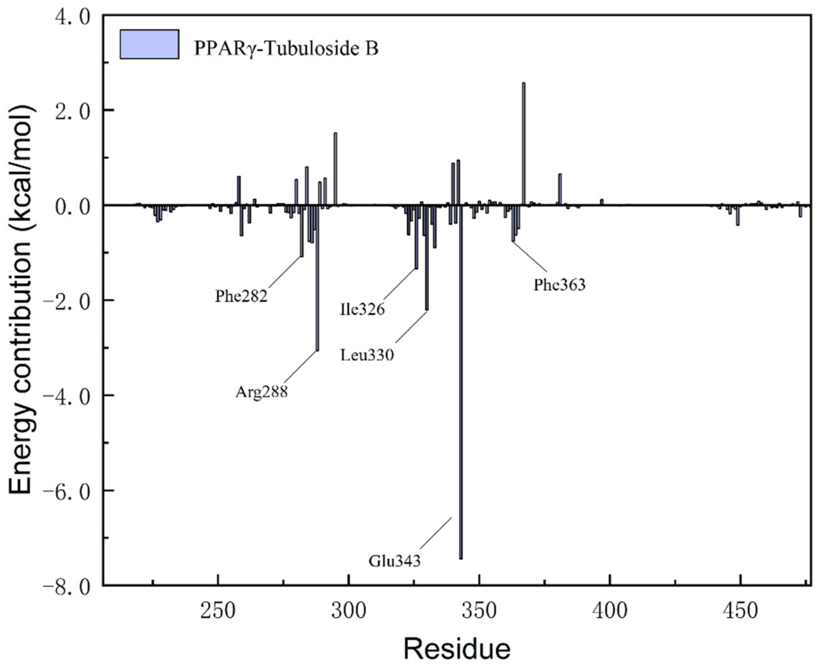

Figure S10. Energy contribution per residue to the binding of PPARγ with tubuloside b.

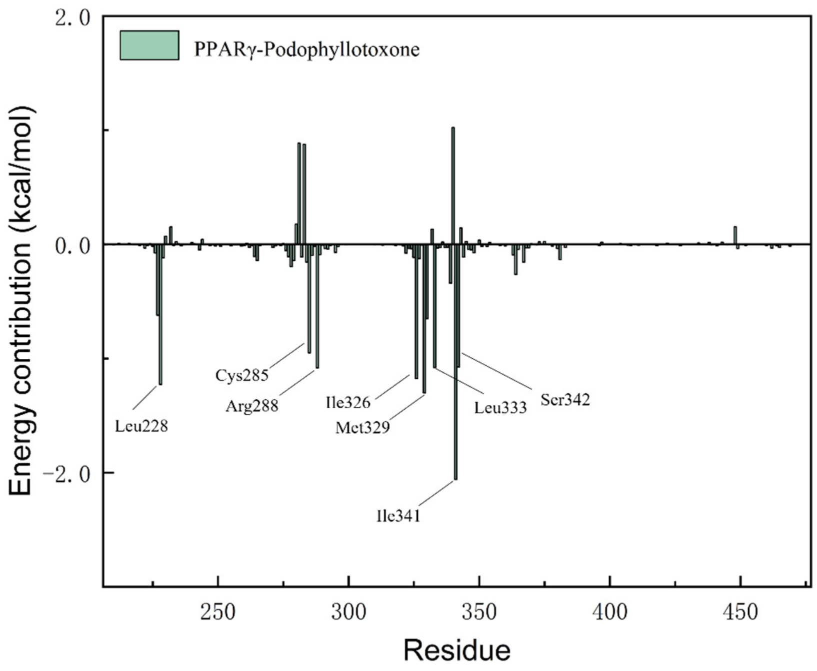

Figure S11. Energy contribution per residue to the binding of PPARγ with podophyllotoxone.

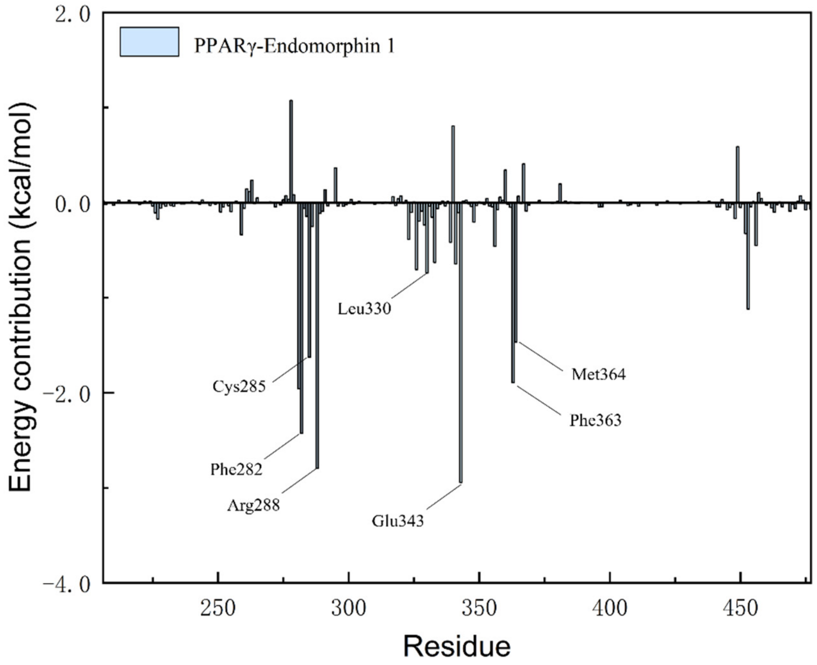

Figure S12. Energy contribution per residue to the binding of PPARγ with endomorphin 1.

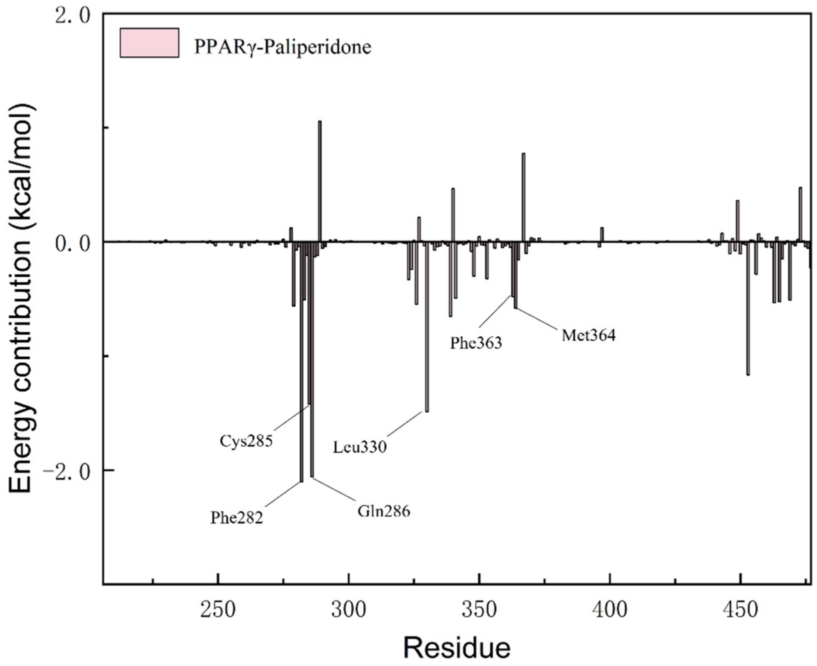

Figure S13. Energy contribution per residue to the binding of PPARγ with paliperidone.
